# Epithelial and microenvironmental mosaicism under stress: a two-compartment model for lung cancer development in never-smokers

**DOI:** 10.3389/fcell.2026.1891974

**Published:** 2026-06-23

**Authors:** Risa Burr

**Affiliations:** Department of Molecular Genetics and Cell Biology, University of Chicago, Chicago, IL, United States

**Keywords:** cancer predisposition, clonal selection, genetic mosaicism, integrated stress response (ISR), lung cancer in never-smokers, tumor microenvironment

## Abstract

The somatic mutation theory frames cancer initiation as the consequence of accumulating driver mutations. However, the widespread presence of oncogenic mutations in normal tissues and the rarity with which these clones progress to malignancy suggest that mutation alone is insufficient. Non-small-cell lung cancer in never-smokers exemplifies this paradox, as tumors often arise in the context of low mutational burden. Here, I integrate insights from developmental mosaicism and stress biology to propose a two-compartment model of cancer development. I argue that epithelial and microenvironmental genetic mosaicism establish latent initiated fields of susceptibility, while environmental and intrinsic stressors—particularly those engaging the integrated stress response—act as selective filters that determine which clones expand, adapt, or remain latent. In this framework, cancer development reflects the convergence of permissive epithelial clones, a supportive or altered microenvironment, and sustained stress. This perspective extends classical somatic evolution and field cancerization models by emphasizing the role of stress-mediated selection across interacting tissue compartments, with implications for early detection and prevention.

## Introduction: beyond mutation as a sufficient driver of cancer development

The somatic mutation theory posits that cancer arises through the progressive accumulation of driver mutations within a cell lineage. While this framework has been foundational, accumulating evidence suggests that it is incomplete. Large-scale sequencing studies have revealed that canonical oncogenic driver mutations are common in histologically normal tissues, including epithelia of the lung, skin, and esophagus (Martincorena and Campbell, 2015; Yokoyama et al., 2019; Yizhak et al., 2019). At the same time, many such clones remain indolent, and pre-neoplastic lesions frequently fail to progress. This creates a central paradox: oncogenic mutations are common, whereas cancer is comparatively rare. This discrepancy suggests that the presence of driver mutations is necessary but not sufficient for cancer development.

Non-small-cell lung cancer (NSCLC) in never-smokers provides a particularly compelling context in which to revisit this model. Approximately 25% of all lung cancer cases worldwide are not attributable to smoking, and no predominant risk factor has been identified as the major cause of NSCLC in non-smokers (Sun et al., 2007; Weeden et al., 2023). These tumors often harbor canonical driver mutations in oncogenes such as EGFR but exhibit relatively low overall mutational burden (Sun et al., 2007; Kadara et al., 2019). Moreover, environmental risk factors such as air pollution appear to promote tumorigenesis through mechanisms that are not primarily mutagenic (Hill et al., 2023). Together, these observations point toward a model in which mutation provides a latent substrate, but cancer development depends on additional selective forces.

In this mini-review, I integrate insights from developmental mosaicism and stress biology to propose a unified framework for cancer development in never-smoker NSCLC. I suggest that inherited or early-acquired genetic alterations establish a mosaic epithelial and microenvironmental landscape, while environmental and intrinsic stress responses—particularly the integrated stress response (ISR)—function as selective filters that convert latent genetic mosaicism into differential clonal fitness.

## Epithelial mosaicism defines latent fields of tumor initiation

Recent work has established that normal tissues are composed of genetically diverse populations of cells, often reflecting accumulation of age-associated mutations. Across tissues, clones harboring mutations in canonical cancer genes—including TP53, NOTCH1, FAT1, and EGFR—can be detected in individuals without cancer (Martincorena and Campbell, 2015; Yokoyama et al., 2019; Curtius et al., 2018). In the esophageal epithelium, for example, aged individuals harbor large numbers of cells with mutations in genes commonly associated with cancer (Yokoyama et al., 2019). Similarly, activating EGFR mutations have been detected in histologically normal lung tissue, including in individuals who never developed lung cancer during life (Hill et al., 2023; Burr et al., 2024).

Developmental mosaicism provides an additional layer of complexity and a direct mechanistic link between genetic predisposition and field cancerization. Post-zygotic acquisition of oncogenic mutations, followed by cellular mixing during organismal development, can generate geographically distinct patches of altered tissue early in life (Curtius et al., 2018). In particular, post-zygotic EGFR mutations can generate spatially distributed epithelial clones early in life, establishing long-lived fields of latent susceptibility (Burr et al., 2024). These findings suggest that genetic predisposition may be encoded not only in the germline but also in early developmental events, shaping the baseline architecture of tissues long before cancer development.

These observations support a model, first introduced by Slaughter et al., in 1953, in which epithelial tissues exist as mosaics of mutant and non-mutant cells, the clonal expansion of which forms what has been described as “fields of susceptibility” or “field cancerization” (Slaughter et al., 1953). The presence of field cancerization increases cancer risk (Curtius et al., 2018). However, the presence of such fields does not reliably predict tumor formation, as it remains exceedingly rare that individuals bearing driver mutations or even clonal expansions will develop malignancy (Yokoyama et al., 2019). Consistently, multiple groups have shown that the numbers and genetic signatures of mutations in normal tissues can approach or, in some contexts, exceed those observed in cancers of the same tissues (Martincorena and Campbell, 2015; Curtius et al., 2018). Even indolent lesions are now appreciated to be frequently observed upon autopsy, suggesting that clonal growth may be constrained to a certain size without additional factors (Yizhak et al., 2019; Weeden et al., 2023).

In this review, I will adopt the terms “initiation” and “promotion” from Swanton and others (Weeden et al., 2023; Coussens and Werb, 2002). Initiation is a process that produces a heritable, potentially advantageous, alteration, such as a driver genetic mutation, in a pre-cancerous cell. Promotion is a mechanism by which non-mutagenic factors confer a selective advantage upon certain initiated cells. Mosaic tissues contain a reservoir of genetically distinct initiated clones whose fate is not predetermined, but instead depends on the selective pressures imposed by the tissue environment. The key question, therefore, is what promoters drive the transition from a stable mosaic to a progressively expanding and malignant clone.

## Environmental stress-mediated promotion of epithelial clones

If epithelial mosaicism defines the initiated substrate for cancer development, environmental and intrinsic stressors define the selective pressures that act upon individual cells to inhibit or promote progression.

Air pollution is one stressor that has emerged as a leading potential risk factor for lung cancer in never-smokers. Recent work has demonstrated that particulate matter (PM) exposure promotes lung tumorigenesis not primarily through mutagenesis, but through inflammatory and microenvironmental remodeling (Hill et al., 2023). In mouse models, PM exposure induces macrophage infiltration and IL-1β signaling, which in turn promotes plasticity and expansion of pre-existing EGFR-mutant epithelial cells. Notably, PM exposure is associated with increased tumor incidence without a corresponding increase in the frequency of activating oncogenic mutations, indicating that it acts as a promoter of clonal expansion rather than an initiator of mutation.

Other environmental stressors experienced during tumor development include physical stressors such as compression, deformation, contact inhibition, tissue wounding, and clonal competition; resource limitations such as hypoxia, ischemia, nutrient deprivation, and growth factor limitation; toxic stresses such as acidosis and reactive oxygen species; immune interactions; and external insults including UV exposure, tobacco smoke, pollution, alcohol, diet, infections, and drug treatments (Weeden et al., 2023; Curtius et al., 2018; Coussens and Werb, 2002; Gatenby and Gillies, 2008). Cell-intrinsic stressors include high rates of protein production, oncogene-induced stress, anoikis, and DNA-damage (Curtius et al., 2018; Ghaddar et al., 2021). Some of these stressors are specific to the cancer condition, whereas others are experienced throughout normal development. A central question is how such diverse stressors are integrated at the cellular level to influence clonal fitness.

Stress responses such as the ISR provide a unifying mechanism by which cells adapt to adverse conditions and gain a selective advantage. The ISR, mediated through phosphorylation of eIF2α and downstream reprogramming of gene expression, allows cells to modulate protein synthesis, promote survival pathways, reorganize the cytoplasm, and alter cell state in response to diverse stressors (Smith and Bartel, 2026). Recent work demonstrates that stress granule proteins such as G3BP actively reinforce ISR-dependent translation programs, selectively enhancing the expression of stress-adaptive transcripts (Smith and Bartel, 2026). In oncogenic contexts, stress signaling pathways such as PERK–eIF2α have been shown to support tumor cell survival under hypoxic, proteotoxic, and metabolic stress (Ghaddar et al., 2021; Bi et al., 2005; Atkins et al., 2013). The ISR is upregulated in many cancers and correlates with decreased overall survival (Ghaddar et al., 2021). Pharmacologic inhibition of the ISR in NSCLC cell lines inhibits outgrowth under nutrient stress (Albert et al., 2019). In KRAS G12D-driven NSCLC models, loss of ISR function results in smaller tumors and prolonged survival, consistent with the increased genotoxic, proteotoxic, and metabolic stress associated with KRAS oncogenic transformation (Ghaddar et al., 2021).

Myriad studies demonstrate that environmental exposures interact with underlying genotypes and cell states to reshape the competitive landscape of epithelial tissues. Unlike many stress-response pathways, the ISR directly couples environmental inputs to translational control and cell-state regulation, positioning it as a key determinant of cellular fitness under stress. This raises the possibility that ISR activity does not merely permit survival but actively biases which clones expand within mosaic tissues. Thus, environmental and intrinsic stressors do not simply damage tissue; they actively select for clones that are adapted to those stresses, linking exposure to clonal expansion.

## Microenvironmental mosaicism and immune dynamics shape the selective niche

While epithelial mutations in initiated cells were long the primary focus of models of cancer development, increasing attention has been paid to the role of the tumor microenvironment (TME) in promoting adaptation to environmental stressors (e.g., through upregulation of angiogenesis). Building on this broader perspective, emerging evidence indicates that the TME is itself genetically heterogeneous and can actively shape shaping tumorigenesis.

Even when considering epithelial mosaicism alone, epithelial cells that harbor oncogenic mutations but have not themselves progressed to tumors can still comprise important components of the TME. In NSCLC in non-smokers, a recent study found that 19% of patients harbored activating EGFR mutations in normal lung tissue that were not present in their tumors (Hill et al., 2023). In some cases, such as NOTCH1 and PPM1D, canonical oncogenic mutations are actually more frequently detected in normal epithelium than in the associated cancer type (Yokoyama et al., 2019). Loss of NOTCH1 in normal mouse skin epithelia is also sufficient to drive non-cell autonomous tumor formation in neighboring NOTCH1-intact cells (Demehri et al., 2009).

Similarly, genetic alterations in non-epithelial TME compartments can promote tumorigenesis. Disruption of Notch signaling through deletion of the CSL/RBP-Jk gene in mesenchymal cells, for example, generates a cancer-associated fibroblast-like state characterized by inflammatory and proteolytic activity, which in turn promotes non-cell autonomous epithelial tumor formation (Hu et al., 2012). Notably, these effects can occur even when the epithelial compartment does not share the same genetic alteration, highlighting the capacity of the microenvironment to drive tumorigenesis through non-cell-autonomous mechanisms.

Clonal hematopoiesis of indeterminate potential (CHIP), characterized by age-associated mutations in hematopoietic stem cells, represents a major source of TME mosaicism. CHIP is associated with an increased mortality in patients with solid tumors, as well as an increased incidence of lung cancer (Pich et al., 2025). Recent studies have shown that CHIP-derived immune cells can infiltrate solid tumors, including approximately one-third of NSCLC tumors, and can also influence disease progression (Pich et al., 2025). For example, TET2-mutant myeloid cells enhance tumor growth in organoid and *in vivo* models, likely through altered inflammatory signaling and cytokine production. Early analyses indicate that the genotype matters, with TET2 mutations serving as an independent positive predictor of tumor infiltration, whereas PPM1D mutations are associated with reduced infiltration (Pich et al., 2025). These findings suggest that the immune compartment is not merely reactive but can be preconditioned by genetic alterations that influence its behavior within tissues.

Together, these observations support a model in which the tumor microenvironment is an active, genetically heterogeneous ecosystem that shapes the selective pressures experienced by epithelial cells. Both epithelial and microenvironmental compartments can independently harbor mosaic genetic alterations, creating a bidirectional system of selection and signaling.

## Discussion: a two-compartment model of cancer development

**FIGURE 1:**
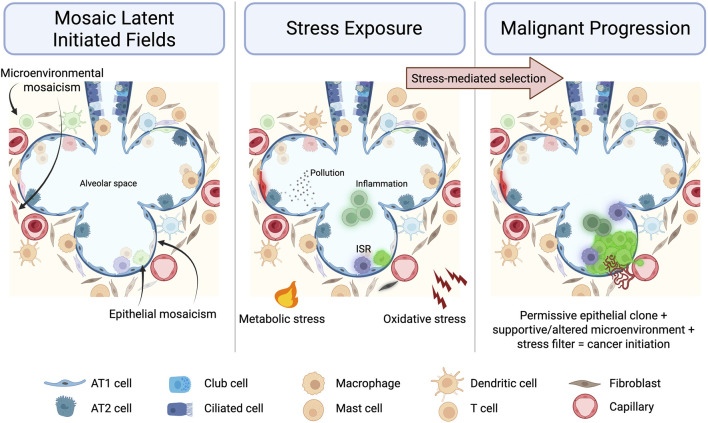
A two-compartment model of stress-mediated lung cancer initiation in never-smokers. Epithelial and microenvironmental mosaicism establish latent fields of initiated cells that are subject to selection by environmental and intrinsic stressors. Left: Normal lung tissue contains genetically heterogeneous epithelial and microenvironmental cell populations arising from developmental, germline, and age-associated processes, forming latent fields of susceptibility. Middle: Environmental and intrinsic stressors—including particulate matter exposure, inflammation, metabolic stress, and oxidative stress—are sensed within the tissue and integrated through stress-response pathways such as the integrated stress response (ISR), reshaping the local microenvironment. Right: Under conditions of sustained stress, selective pressures favor the expansion of stress-adapted epithelial clones within permissive or altered microenvironments, leading to malignant progression. In this framework, cancer initiation reflects the convergence of a permissive epithelial clone, a supportive or altered microenvironment, and stress-mediated selection.

I propose that cancer development in never-smoker lung cancer is best understood as the convergence of three factors: epithelial and microenvironmental mosaicism, arising through inherited, developmental, and age-associated processes; environmental and intrinsic stressors, including inflammatory signaling and activation of stress-response pathways such as the ISR; and a dynamic tissue ecosystem in which these forces interact to shape clonal fitness (Figure 1).

In this model, mosaicism defines a latent landscape of susceptibility, while stress responses act as selective filters that determine which clones expand, adapt, or regress. Cancer development occurs when an initiated epithelial clone is promoted through the combined actions of a supportive or altered microenvironment and conditions of sustained stress.

Importantly, latent genetic susceptibility is not solely a theoretical construct, but is directly observed in patients through both germline predisposition and developmental mosaicism. Early post-zygotic acquisition of oncogenic mutations, followed by cellular mixing during development, can generate geographically distinct patches of altered tissue, contributing to epithelial and/or microenvironmental mosaicism depending on the timing of mutation (Burr et al., 2024). In parallel, germline mutations in cancer-associated genes—including BRCA1, TP53, and EGFR—establish organism-wide alterations across both epithelial and microenvironmental lineages. Notably, even heterozygous loss of tumor suppressors such as BRCA1 is associated with altered tumor microenvironments, including reduced CD8^+^ tumor-infiltrating lymphocytes in breast tumors compared to wild-type controls (Wu et al., 2023). Together, these observations position cancer predisposition and developmental mosaicism upstream of stress-mediated selection, defining a temporal axis from early-life mutation to adult tumorigenesis.

This framework extends, rather than replaces, existing models. Classical somatic mutation theory correctly identifies driver mutations as necessary components of tumor initiation but does not explain why such mutations are frequently observed in normal tissues without progression. Similarly, clonal evolution models describe mutation-selection dynamics but often treat the selective landscape as static. Here, we emphasize that the selective landscape itself is dynamic, shaped by stress and microenvironmental context. Field cancerization models recognize the existence of altered but nonmalignant tissue fields; however, they typically focus on the epithelial compartment. Here, I extend this concept by proposing that the relevant field spans at least two interacting compartments—epithelial and microenvironmental—whose crosstalk under stress governs cancer development.

Therefore, this model differs from prior frameworks in three key ways.

- It treats mutation as generating latent heterogeneity rather than deterministic drivers of cancer;
- It incorporates dynamic, stress-dependent fitness landscapes;
- It extends field cancerization to include genetically and functionally heterogeneous microenvironmental compartments.

This framework has potential therapeutic implications. If cancer development depends on stress-mediated selection of latent fields, then interventions that modulate inflammatory signaling, stress-response pathways such as the ISR, or the composition of the microenvironment may alter the trajectory of pre-neoplastic fields. For example, targeting IL-1β signaling or modulating macrophage recruitment could reduce the expansion of high-risk epithelial clones in response to environmental exposures (Hill et al., 2023). Similarly, understanding how stress-response pathways influence clonal fitness may reveal opportunities to selectively disadvantage pre-neoplastic cells. More broadly, this framework suggests that cancer risk may be better predicted by the interaction between genetic mosaicism and stress exposure, rather than either factor alone. It further implies that cancer prevention strategies may benefit from targeting not only mutation accumulation, but also the ecological and stress-driven dynamics that govern clonal selection.
